# Application of Near-Infrared Spectroscopy for Understanding Spontaneous Brain Activity During Resting State in Schizophrenia: A Mini Review

**DOI:** 10.3389/fpsyt.2021.704506

**Published:** 2021-08-13

**Authors:** Masaya Yanagi, Osamu Shirakawa

**Affiliations:** Department of Neuropsychiatry, Kindai University Faculty of Medicine, Osakasayama, Japan

**Keywords:** NIRS, resting-state, spontaneous brain activity, medial prefrontal cortex, ALFF, fALFF, schizophrenia

## Abstract

Spontaneous brain activity occurs at rest, as represented by the default mode network. A resting paradigm is suitable for investigating brain function of patients with psychiatric diseases who may have difficulties adhering to goal-oriented tasks. Evidence accumulated in neuroimaging studies using functional magnetic resonance imaging has shown that the resting cerebral blood flow is impaired in psychiatric diseases. Near-infrared spectroscopy (NIRS), a simple neuroimaging modality, is an optimal tool for the resting paradigm, because it can offer a comfortable environment for measurement. Recent NIRS studies have demonstrated some promising data of altered resting activity in the prefrontal cortex of patients with schizophrenia, which may be exploited to develop further applications of NIRS in clinical psychiatry. Based on these findings, we emphasize the benefits of NIRS for assessing the prefrontal pathophysiology during the resting state and some methodological issues to be noted while analyzing cerebral blood flow using NIRS; moreover, we focus on interpreting these changes based on the complex nature of the spontaneous brain activity during resting state.

## Introduction

While numerous lines of biological evidence have accumulated to support the pathophysiology of psychiatric diseases, a limited amount of the evidence is available to guide clinical management of these diseases. In order to effectively integrate the evidence from research into clinical practice, the translational aspects of biological evaluations in psychiatry need to be considered. In a trial undertaken in Japan, a simple neuroimaging modality, near-infrared spectroscopy (NIRS), was employed with the purpose of assisting the differential diagnoses of patients who manifested depressive symptoms. In this trial using NIRS, the prefrontal blood flow of the patients during a verbal fluency task was employed to distinguish major depression, bipolar disorder, and schizophrenia (1). This simple assessment approach was successfully trialed in psychiatric clinics in Japan. However, the reported sensitivity of this assessment approach of around 60% remains a limitation (2). This critical issue was partially addressed in a previous study, which reported a difficulty to biologically delineate the boundary between schizophrenia and bipolar disorder when categorized based on current clinical diagnostic criteria (3). A review on this assessment approach raises some technical issues also, such as a variability in the performance of verbal fluency task among patients, which could be a confounding factor in analyzing prefrontal blood flow changes (4). To overcome these technical issues, a resting paradigm may be beneficial. A resting paradigm is readily accessible even for patients who have some difficulties in performing goal-oriented tasks. In addition, NIRS is suitable for a resting paradigm, because it can offer a quiet approach to neurophysiological measurements. NIRS is suitable for clinical settings on the basis of its simplicity. In this article, we outline the potential of NIRS to evaluate prefrontal pathophysiology with a resting paradigm, using cases of schizophrenia as an example.

## Spontaneous Brain Activity During Resting State

Spontaneous brain activities occur during resting state. A specific feature of these activities is the activation of several brain regions during resting state, which is termed the default mode activity (5). The major brain regions where a default mode activity has been observed are the medial prefrontal cortex (mPFC), posterior cingulate cortex (PCC)/precuneus, inferior parietal lobule, and lateral temporal cortex (6, 7). This cluster of brain regions is called the default mode network (6, 7). Within the default mode network, the medial prefrontal cortex (mPFC) and PCC/precuneus are the core regions of this network that are constantly activated by any activities of the default mode network (5, 7, 8).

The other specific feature of the brain activity during resting state is the spontaneous low-frequency (<0.1 Hz) fluctuations of the cerebral blood flow (9). These fluctuations are synchronized among brain regions within the default mode network. By characterizing the simultaneous low-frequency (<0.1 Hz) fluctuations of the cerebral blood flow among brain regions in the resting paradigm, functional magnetic resonance imaging (fMRI) studies have identified various intrinsic brain functional networks other than the default mode network, such as the salience network and the executive network (9, 10). Furthermore, recent studies using brain functional network analysis have reported disease subtype-specific network impairments that may address future therapeutic interventions for psychiatric diseases (11–13). As such, resting-state neuroimaging is a promising tool that could be applied for the biological evaluation of psychiatric disorders.

## Default Mode Activity in Schizophrenia

Accumulating evidence in fMRI studies has shown that the default mode network is impaired in schizophrenia (14–16). The initial research for the default mode network has elucidated the task-induced deactivation failure of the default mode network in patients with schizophrenia using a subtraction method that quantifies the difference of the activity between the resting state and the condition while performing cognitive tasks (17–19). Further studies of the default mode network have focused on analyzing the activity during extended task-free rest periods using the resting paradigm. The task-free paradigm has merit for the examination of psychiatric diseases, because it is easily applied to patients who may have difficulties adhering to goal-oriented tasks. In this paradigm, brain functional network research uses time domain analysis for low-frequency fluctuations of cerebral blood flow through examining the brain regional correlations during the extended rest periods. In addition, the intensity of the brain activity during resting state has been evaluated by analyzing the frequency domain of low-frequency fluctuations of regional cerebral blood flow.

One of the methods to quantify the intensity of brain activity in the resting paradigm is known as the amplitude of low-frequency fluctuations (ALFF). ALFF is calculated as the sum of the power across low-frequency ranges, such as 0.01–0.08 Hz, of cerebral blood flow fluctuations (20). In addition to the ALFF, fractional ALFF (fALFF), the ratio of the ALFF to the total power, is also used as an indicator that may receive less noise from physiological sources compared to the ALFF (21). Both ALFF and fALFF capture hemodynamic signals from the gray matter of brain, prominently in the core region of default mode network, namely, mPFC and PCC/precuneus (20, 21). Several studies examining ALFF or fALFF using the resting paradigm have shown that they are reduced in mPFC and PCC/precuneus in patients with schizophrenia (22–29). These findings suggest that the core region of the default mode network has diminished activity during the resting state in schizophrenia.

## Application of NIRS for Measuring the Brain Activity in the Rest Period

NIRS is a simple neuroimaging modality that is easy to use and offers non-invasive measurement. Details of the NIRS system have been described in previous reviews (30, 31). This system allows the measurement of cerebral blood flow without any acoustic scanner noises, which are inevitable when performing fMRI measurements. Additionally, the measurements with NIRS require fewer physical constraints, and hence, it is tolerant to small movements. Because of these benefits, it offers a relatively comfortable environment to the examinee, and thus, NIRS is an optimal modality to apply to the resting paradigm. In addition, test and retest studies have verified the stability of NIRS measurements for detecting brain networks during resting state (32–34). Given that the resting paradigm is easily adopted by patients with psychiatric diseases, NIRS is suitable for assessing the brain activity of these patients during resting state. Here, we will describe some technical issues relating to NIRS in interpreting hemodynamic signals. Following this, we will introduce NIRS studies of schizophrenia that examined brain activity using a resting paradigm and discuss the complex nature of spontaneous brain activity during resting state that is present in healthy subjects.

### Methodological Issues in Interpreting NIRS Signals

While the NIRS system has advantages in terms of its simplicity and economical efficiency, it has some technical limitations that need to be noted when analyzing the cerebral blood flow. A major limitation is that NIRS signals lack precise information to detect cortical activity. NIRS measures hemodynamic signals up to only a cortical surface at a depth of 2–3 cm from the skin (35–37) by using an infrared source and detector probes that are chiefly set on the forehead (30, 38). Because the NIRS signals are scattered near-infrared light signals that are emitted from the source probe and caught by a detector probe, the spatial resolution of the NIRS channel defined by these probes is not enough to annotate the hemodynamic signals of the precise cortical regions. In addition, NIRS signals include contamination of peripheral hemodynamic factors such as skin perfusion, even in the limited frequency range of blood flow fluctuations for ALFF (0.01–0.08 Hz) (39). However, a previous study analyzing the cerebral blood flow during resting state with simultaneous measurements of NIRS and fMRI demonstrated that the NIRS signals detected in most of the channels were highly correlated with the signals of brain tissue in the fMRI measurement (40). Taken together, this suggests that NIRS detects the hemodynamic signals that reflect the cortical activity in roughly segmented cortical area, although the influence of signals from the peripheral tissues cannot be fully excluded.

### Complex Nature of the Brain Activity During Resting State

**Figure 1:**
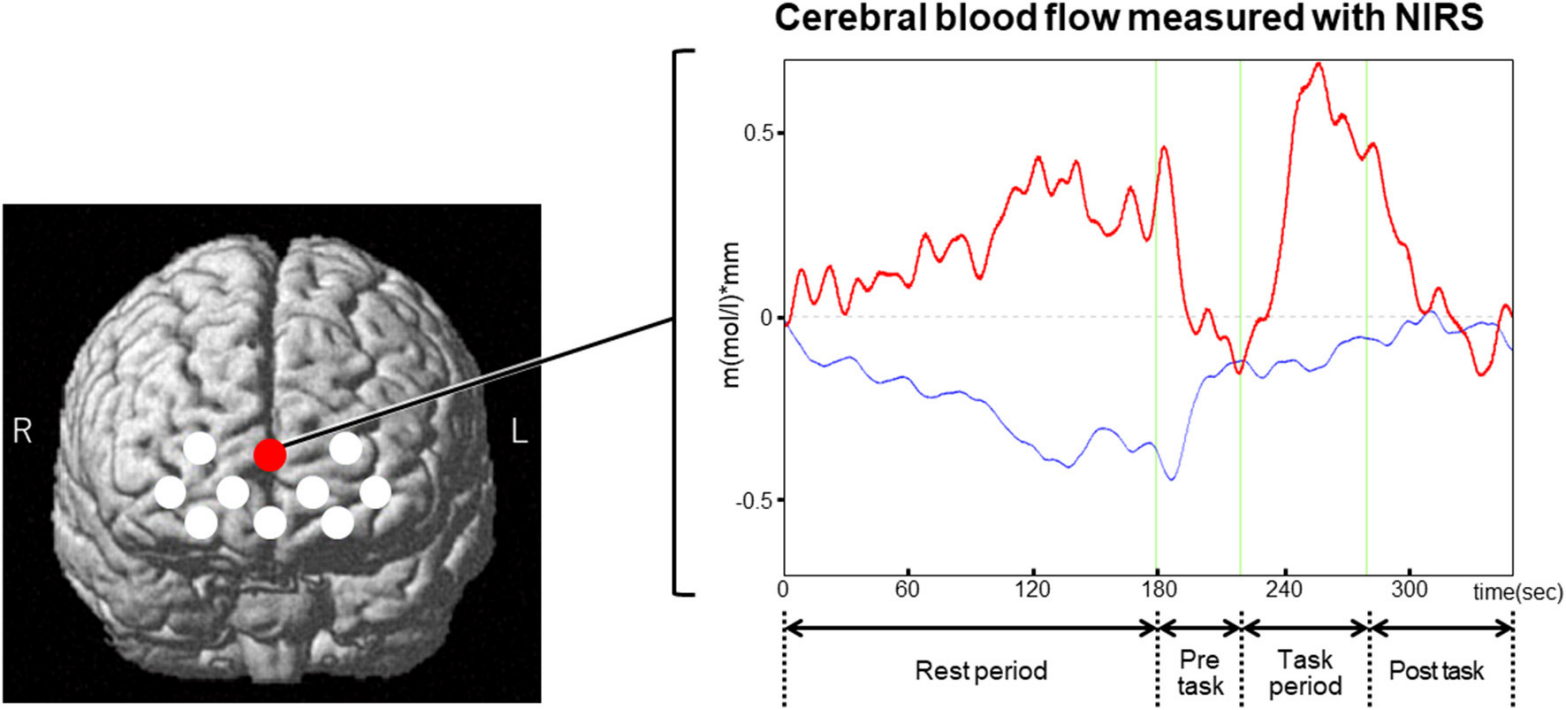
An example of NIRS measurement using a resting paradigm that was followed by a verbal fluency task. The left figure displays NIRS channel locations that are indicated by the white and red circles. The red circle points to the channel that displays an example (right figure) for the time course of NIRS signals in a healthy subject. In this case, the NIRS signals were potently activated during the rest period. The activation of the signals was reversed during the subsequent pre-task, where the subject simply repeated a sequence of syllables (a, i, u, e, o). The NIRS signals were activated again during the task period for the verbal fluency task, which asks the subject to generate as many words as possible starting with presented syllables such as “a.” The activation of the signals induced by the verbal fluency task was reversed during the post-task, which consisted of the same task as the pre-task. The magnitudes of the activated signals during the rest period were nearly comparable with those induced by the verbal fluency task. This potent activation of the NIRS signals may indicate some spontaneous cognitive activity that occurred during the rest period. Red bold line, oxy-hemoglobin signal; blue line, deoxy-hemoglobin signal.

The discovery of default mode activity was prompted by the observation that our brain is not resting when we are resting (7). Although the default mode network was termed for the activated brain regions at rest, this network is more active in the tasks demanding self-referential mental activities (6–8). Furthermore, it is known that the spontaneous cognition toward internal mentation such as self-referential mental activity often occurs during resting state (6, 7). These lines of evidence suggest that a high variability of brain activation could be generated during resting state depending on one's own spontaneous cognitive status. Figure 1 displays a NIRS measurement in a channel located on the superficial part of mPFC in a healthy subject. The NIRS signals demonstrate a potent activation during resting state, which are nearly comparable with those induced by the following verbal fluency task. This potentiation of the brain activity may reflect some ongoing spontaneous cognitive activity that occurred in the rest period. As suggested in this case, spontaneous cognitive activities could occur in the resting paradigm. These spontaneous activities may hamper the attainment of a stable baseline of brain activity during resting state for each subject. Such a complex nature of brain activity during resting state needs to be noted in interpreting changes of cerebral blood flow during the extended rest period.

### Examining Brain Activity With NIRS During Resting State in Schizophrenia

Recent studies have demonstrated some promising findings with NIRS to capture the alteration of frontal activity during resting state in schizophrenia. An initial study using two-channel NIRS reported a reduction of the frontal blood flow in the rest period in patients with chronic schizophrenia (41). Recent studies using NIRS whose channels cover the entire forehead demonstrated that the magnitude of brain activation as well as ALFF and fALFF were decreased during resting state in the channels located around the mPFC region in patients with chronic schizophrenia (42, 43). Given that mPFC is a core brain region of the default mode network where spontaneous brain activities occur during resting state in healthy subjects depending on their spontaneous cognitive state, these results suggest that the spontaneous activities were deficient in the patients with chronic schizophrenia. That is to say, the decreased activity during resting state that was observed in the vicinity of mPFC in chronic schizophrenia may represent the impaired ability of patients to produce and maintain the function of the default mode network such as the self-referential mental activity. Based on these findings, we emphasize the benefits of NIRS for assessing the prefrontal pathophysiology of schizophrenia using a resting paradigm. Moreover, the benefits of NIRS for the resting paradigm could be exploited to develop further applications of this modality not only for schizophrenia but also for other psychiatric diseases. Although resting-state NIRS findings are only in the early stages of application in psychiatry, the resting-state NIRS may develop valid biomarkers adjunctively with other neuromodalities to offer some elucidation on the prefrontal pathophysiology of the psychiatric diseases.

## Future Direction

This review focused on changes in regional spontaneous brain activity within limited reports of the resting-state NIRS for patients with schizophrenia. Considering that this technique also enables the assessment of functional brain connectivity, further investigations with NIRS using the resting paradigm should be explored in patients with schizophrenia. Previous NIRS studies using the resting paradigm have reported impairments in functional brain connectivity in patients with major depression (44–46). These features of NIRS with the resting paradigm encourage further applications of the technique to differentiate among major psychiatric disorders, including schizophrenia, based on pathophysiology. Cross-diagnostic studies using NIRS with the resting paradigm may contribute to the identification of disease-specific or disease subtype-specific changes among these disorders. The changes observed in resting-state NIRS studies could be further developed by applying this simple assessment system in daily psychiatric clinical practice. These challenges are inherent in the search for a biomarker-driven classification of psychiatric disorders, which may address the heterogeneity of these disorders and provide a baseline for future biomarker-driven therapeutics in psychiatry.

## Conclusion

In summary, NIRS is a valid tool to assess the spontaneous brain activity during resting state. Previous studies have provided some promising evidence that NIRS could detect the disease-associated alteration of prefrontal activity in a resting paradigm in schizophrenia. Although some methodological limitations of NIRS and the complex nature of resting brain activity need to be noted while interpreting NIRS signals, the simplicity of NIRS in relation to a resting paradigm is advantageous for routine applications in clinical psychiatric settings. Further clinical applications of NIRS should be explored with a resting paradigm to develop a biomarker that may address future therapeutic interventions for psychiatric diseases.

## Author Contributions

MY wrote the manuscript and OS edited it. Both authors have read and approved the final manuscript.

## Conflict of Interest

The authors declare that the research was conducted in the absence of any commercial or financial relationships that could be construed as a potential conflict of interest.

## Publisher's Note

All claims expressed in this article are solely those of the authors and do not necessarily represent those of their affiliated organizations, or those of the publisher, the editors and the reviewers. Any product that may be evaluated in this article, or claim that may be made by its manufacturer, is not guaranteed or endorsed by the publisher.
